# Thermal and Bulk Properties of Triblock Terpolymers and Modified Derivatives towards Novel Polymer Brushes

**DOI:** 10.3390/polym15040848

**Published:** 2023-02-08

**Authors:** Konstantinos Artopoiadis, Christina Miskaki, Gkreti-Maria Manesi, Ioannis Moutsios, Dimitrios Moschovas, Alexey A. Piryazev, Maria Karabela, Nikolaos E. Zafeiropoulos, Dimitri A. Ivanov, Apostolos Avgeropoulos

**Affiliations:** 1 Department of Materials Science Engineering, University of Ioannina, University Campus-Dourouti, 45110 Ioannina, Greece; 2 Institut de Sciences des Matériaux de Mulhouse—IS2M, CNRS UMR7361, 15 Jean Starcky, 68057 Mulhouse, France; 3 Institute of Problems of Chemical Physics, Russian Academy of Sciences, Chernogolovka, 142432 Moscow, Russia; 4 Faculty of Chemistry, Lomonosov Moscow State University (MSU), GSP-1, 1-3 Leninskiye Gory, 119991 Moscow, Russia

**Keywords:** low molecular weight triblock terpolymers, hydroboration/oxidation reactions, modification of PB_1,2_, high *χ* terpolymers, self-assembly, asymmetric interactions, double gyroid, polymer brushes, wettability

## Abstract

We report the synthesis of three (3) linear triblock terpolymers, two (2) of the ABC type and one (1) of the BAC type, where A, B and C correspond to three chemically incompatible blocks such as polystyrene (PS), poly(butadiene) of exclusively (~100% vinyl-type) -1,2 microstructure (PB_1,2_) and poly(dimethylsiloxane) (PDMS) respectively. Living anionic polymerization enabled the synthesis of narrowly dispersed terpolymers with low average molecular weights and different composition ratios, as verified by multiple molecular characterization techniques. To evaluate their self-assembly behavior, transmission electron microscopy and small-angle X-ray scattering experiments were conducted, indicating the effect of asymmetric compositions and interactions as well as inversed segment sequence on the adopted morphologies. Furthermore, post-polymerization chemical modification reactions such as hydroboration and oxidation were carried out on the extremely low molecular weight PB_1,2_ in all three terpolymer samples. To justify the successful incorporation of –OH groups in the polydiene segments and the preparation of polymeric brushes, various molecular, thermal, and surface analysis measurements were carried out. The synthesis and chemical modification reactions on such triblock terpolymers are performed for the first time to the best of our knowledge and constitute a promising route to design polymers for nanotechnology applications.

## 1. Introduction

The ability of polymers to self-assemble in bulk or thin films using solvents with different selectivity enables the formation of various well-defined morphologies at the nanoscale. This fundamental characteristic is of major importance, leading to their possible utilization in nano-lithographic applications as reported in the literature [1,2,3]. 

Triblock terpolymers adopt various ordered three-phase morphologies not evident in linear diblock and triblock copolymers as expected due to the lack of the third segment. It is documented that the morphologies in ABC systems are significantly affected by the block sequence (ABC vs. BCA vs. CAB), the volume fraction ratios, and the three different values of the Flory–Huggins interaction parameters (*χ*_AB_, *χ*_BC_, *χ*_BA_) [4,5,6]. Unique pattern geometries are adopted after solution and/or spin casting methods, including nanoscale rings that provide electronic [7,8,9,10], optical [11,12,13,14], and magnetic [15,16] properties that are highly desirable for several applications. Despite this, due to the dissimilar properties of the adjacent segments, such as solubility and interfacial or surface energy, only limited research has been conducted in this field.

To tune the self-assembly and surface properties, the scientific community has shifted its interest towards polymer brushes [17,18,19,20,21,22,23,24,25,26,27], which are comprised of a polymeric backbone with attached active sites that may provide the desired surface properties [18]. A route to obtaining specific surface properties is to combine different segments bearing functional groups and then perform post-polymerization modification reactions. The modified segment plays the polymer brush role. Note that polydienes constitute a class of materials that can be easily modified, as already reported in the literature [28,29,30].

ABC and BAC terpolymers consisting of polystyrene or PS (A), polybutadiene of exclusively (~100%) -1,2 microstructure or PB_1,2_ poly(dimethylsiloxane) or PDMS (C) showcase promising potential for pattern fabrication. PS and PDMS are highly immiscible blocks, leading to a high *χ* parameter and, therefore, extremely low dimensions. Their utilization in film formation for different applications has been thoroughly elaborated [31,32,33,34,35,36], but the dissimilar surface energies between the vinyl and siloxane segments favor the parallel orientation. To overcome this limitation, several strategies have been employed, such as the use of homopolymer brushes (hydroxyl-terminated PS and/or PDMS), pre-patterned surfaces, solvent annealing, etc. [1,37,38,39,40,41]. 

New properties are emerging by incorporating an additional low molecular weight segment, namely poly(butadiene). The role of poly(butadiene) of exclusively (~100% vinyl-type) -1,2 microstructure is dual:

(a). Due to the complexity of the system, very low domain periodicities for the PB domains can be formed without miscibility constraints.
(b). Poly(butadiene) can be chemically modified leading to the addition of the -OH functional group in each PB monomeric unit, making the modified PB a sacrificial segment during the film formation, a fact that renders the use of homopolymer brushes unnecessary.

To the best of our knowledge, the synthesis of such terpolymers, their chemical modification, and their self-assembly properties are novel discoveries that have not yet been published in the literature. 

Taking into consideration the previous study conducted by Avgeropoulos’ group [41] where, among others, the bulk properties of PS-*b*-PB_1,4_-*b*-PDMS or PB_1,4_-*b*-PS-*b*-PDMS were reported, it is straightforward that low molecular weight triblock terpolymers are not frequently encountered in the relative literature and the importance of high χ/low N (N: degree of polymerization) terpolymers in microelectronics could be proven most vital.

Herein, we report the synthesis, characterization, and post-polymerization chemical modification reactions of PS-*b*-PB-*b*-PDMS and PB-*b*-PS-*b*-PDMS terpolymers where the PB is exclusively (~100% vinyl-type) -1,2 microstructure. In total, three (3) samples were synthesized (two of the PS-*b*-PB_1,2_-*b*-PDMS sequence and one of the PB_1,2_-*b*-PS-*b*-PDMS) using anionic polymerization and sequential addition of the three monomers. Note that only through anionic polymerization can such terpolymers with specific molecular characteristics be synthesized. Based on the sub-10 nm requirements, the total average molecular weight values (${\bar{M}}_{n}$) for the terpolymers were chosen to be in the range of 9 kg/mol to 18 kg/mol, while the ${\bar{M}}_{n}$ of the PB_1,2_ did not exceed 4 kg/mol in any case.

The molecular characterization of the synthesized samples was accomplished via size exclusion chromatography (SEC), vapor pressure osmometry (VPO), and proton nuclear magnetic resonance spectroscopy (^1^H-NMR), indicating the synthesis of well-defined, narrowly dispersed terpolymers. In addition, differential scanning calorimetry (DSC) experiments were carried out to determine the characteristic thermal properties of the involved segments. The morphological characterization of all unmodified samples in bulk was performed with transmission electron microscopy (TEM) and small angle X-ray scattering (SAXS), revealing the formation of well-developed structures with dimensions as low as 16 nm in certain cases.

Also, the post-polymerization chemical modification reactions of the PB_1,2_ block are reported, and additional characterization in bulk and solution was employed to evaluate the incorporation of –OH groups in all polydiene vinyl-type monomeric units. The successful chemical modification was verified through thermal analysis (thermogravimetric analysis, or TGA and DSC), infrared spectroscopy, ^1^H-NMR, and contact angle measurements—before and after post-treatment for comparison reasons.

## 2. Materials and Methods

### 2.1. Materials

The purification procedures of the involved reagents, including solvents [benzene (99%), tetrahydrofuran (99%)], monomers [styrene (99%), hexamethylcyclotrisiloxane (D_3_) (98%), 1,3-butadiene (99%)], termination agent [methanol (98%)], 1,2-dipiperidinoethane [dipip (98%)], and initiator [*secondary*-BuLi (*sec*-Buli, 1.4 M in cyclohexane)] are documented elsewhere [29,41]. 9-borabicyclo[3.3.1]nonane or 9-BBN (0.5 M in tetrahydrofuran), hydrogen peroxide (H_2_O_2_) and sodium hydroxide (NaOH) were used as received. All reagents were supplied by Sigma-Aldrich (Sigma-Aldrich Co., St. Louis, MO, USA).

#### 2.1.1. Synthesis of PS-*b*-PB_1,2_-*b*-PDMS

**Scheme 1:**
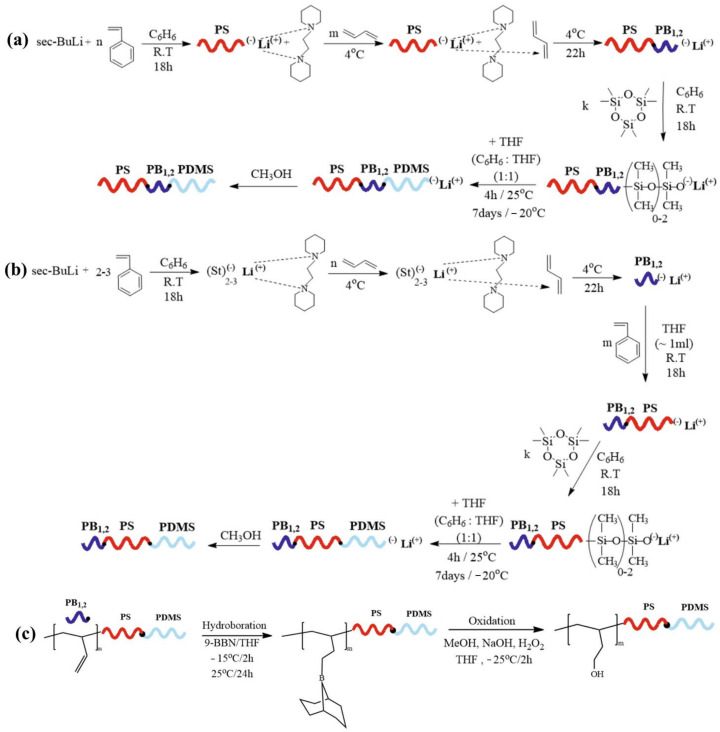
Synthetic routes corresponding to: (**a**) the synthesis of triblock terpolymers of the PS-*b*-PB_1,2_-*b*-PDMS type through anionic polymerization; (**b**) the synthesis of PB_1,2_-*b*-PS-*b*-PDMS triblock terpolymers through anionic polymerization; and (**c**) post-polymerization chemical modification reactions, including hydroboration and oxidation of the PS-*b*-PB_1,2_-*b*-PDMS terpolymers. Note that similar chemical modification reactions are performed in the case of PB_1,2_-*b*-PS-*b*-PDMS.

Styrene (3.5 g, 33.6 mmoles) reacted with *sec*-BuLi (1.0 mmole) in the presence of benzene for 18 h at room temperature. A small quantity was retracted from the reactor to specify the molecular characteristics of the first segment. Subsequently, 1,2-dipiperidinoethane (3.0 mmoles) was introduced to increase the polarity of the solution prior to the addition of the 1,3-butadiene (1.5 g, 27.7 mmoles). The use of the polar compound results in only (~100%) 1,2-microstructure. The reaction was left to proceed at 4 °C for 22 h, and a second aliquot was retrieved to determine the molecular characteristics of the diblock precursor. Then, hexamethylcyclotrisiloxane (D_3_, 4g, 54.0 mmoles) was introduced and allowed to react for 18 h at ambient conditions. After the ring opening of the cyclic monomer was achieved, tetrahydrofuran was added to the solution, and the reaction proceeded for 4 h at room temperature and then was placed at −20 °C for seven days under continuous stirring. The final triblock terpolymer was precipitated in a non-solvent (methanol) and vacuum-dried. The described quantities correspond to sample 1 (see Table 1). Alternations on the quantity of monomers and initiator led to the preparation of sample 2. The schematics concerning the chemical reactions are given in Scheme 1a.

#### 2.1.2. Synthesis of PB_1,2_-*b*-PS-*b*-PDMS

To synthesize the specific sequence, a slightly different synthetic route was employed from the one previously described. A few oligomeric units of styrene and *sec*-BuLi (0.14 mmoles) were introduced at ambient conditions to the reactor with benzene, enabling the formation of a nucleophile macroinitiator. Then, 1,2-dipiperidinoethane (0.30 mmoles) was added to promote the addition exclusively between the first and second carbon atoms of the 1,3-butadiene (2 g, 37.0 mmoles). The monomer was distilled in the apparatus, and the solution was placed at 4 °C for 22 h until complete consumption of the 1,3-butadiene. A small quantity of polar solvent (THF, ~1 mL) was introduced to change the reaction kinetics. Following that, styrene (3.5 g, 33.6 mmoles) was added and allowed to react at room temperature for 18 h until complete conversion, as already stated. The third monomer (D_3_, 4.5 g, 60.0 mmoles) was introduced into the solution in a process similar to the one previously described. In each synthetic step, small aliquots were taken to study the molecular characteristics. The synthetic routes employed for the preparation of the specific triblock terpolymers are presented in Scheme 1b.

#### 2.1.3. Chemical Modification Reactions of PB_1,2_

To obtain –OH functional end groups, the poly(butadiene) of exclusively (~100%) -1,2 microstructure is submitted to post-polymerization chemical modification reactions, namely hydroboration and oxidation. In this process, each vinyl bond (-CH=CH_2_ per monomeric unit) is converted to -CH_2_-CH_2_-OH, leading to the desired -OH group in all monomeric units of the PB blocks. To perform the hydroboration reaction 0.15 g (3.3 mmol, 6.6 mL) of 9-BBN were introduced into 1 g of PS-*b*-PB_1,2_-*b*-PDMS (sample 1, corresponding to 2.7 mmoles of the PB_1,2_ segment) which was dissolved in tetrahydrofuran (0.2 *w*/*v*%) under nitrogen atmosphere at −15 °C. The solution was left to warm up to ambient conditions and was stirred for 24 h to ensure the completion of the hydroboration process. Notably, the 9-BBN is used in ~20% excess compared to the mol of the PB_1,2_ block. Following this, the solution was placed at −25 °C, and 1 mL of properly degassed methanol was added to deactivate all the excess of the borane reagent. After 30 min, NaOH (3.1 mmoles 6 N, 10% excess compared to the borane moles) was introduced to prevent the development of crosslinked networks due to borane by-products. Following, the oxidizing agent H_2_O_2_ (6.2 mmoles 30% *w*/*v* solution, 50% excess compared to the NaOH moles) was added, and the solution was stirred at −25 °C for 2 h before being placed at 55 °C for 1 h, where the separation of the desired organic and aqueous phases occurred. The organic phase was poured into a 0.25 M NaOH solution, and subsequently, the polymer was dissolved in THF/MeOH and washed with 0.25 M NaOH twice using a Buhner funnel [28,29]. Finally, the polymer was thoroughly washed with copious amounts of distilled water and placed in a vacuum oven to remove any volatile compounds. Coherent procedures were employed in all terpolymers. The chemical modification reactions for sample PB_1,2_-*b*-PS-*b*-PDMS or sample 3 are provided in Scheme 1c. Similar reactions are followed for the other sequence of PS-*b*-PB_1,2_-*b*-PDMS samples.

### 2.2. Methods

The instrumentations with which molecular characterization experiments were performed, including SEC, VPO, ^1^H-NMR, and FT-IR, have already reported in our previous works [30,41].

The thermal characterizations through DSC were carried out in an N_2_ atmosphere using aluminum pans (Tzero^®^, TA Instruments Ltd., Leatherhead, UK) in a TA Instruments Q20 DSC (TA Instruments Ltd., Leatherhead, UK) with a heating/cooling rate of 10 K/min. Thermogravimetric analysis was conducted in a Perkin Elmer Pyris-Diamond instrument (PerkinElmer, Inc., Waltham, MA, USA). The samples (~10 mg) were heated from 40 °C to 600 °C with a heating rate of 5 °C/min under a nitrogen atmosphere.

The wetting properties were studied in a contact angle (OCA 25, DataPhysics Instruments GmbH, Filderstadt, Germany) instrument. The samples were dissolved in different solvents (toluene for the pristine materials and a cosolvent mixture of THF/MeOH in a ratio of approximately 2:1 for the modified ones), preparing 1 wt% solution concentrations, and were then spin coated onto silicon wafers at ambient conditions under specific conditions (3750 rpm for 30 s), leading to films with ~40–50 nm thickness. Silicon wafers were first treated with piranha solution (sulfuric acid/hydrogen peroxide: 3/1), leading to purified substrates. In the measurements, 4 μL droplets of DI water were deposited at a rate equal to 0.5 μL/s. Three different measurements in three regions of the same wafer were conducted, and the average value was calculated and presented in the relative results. The deviation in all cases did not exceed ±2°, indicating the consistency of the results due to the uniform film deposition.

The specifics regarding TEM and SAXS measurements are provided elsewhere [42]. The triblock terpolymers were cast from a dilute solution in toluene (5 wt%), and the solvent evaporation was completed in approximately 7 days. Thin sections of the as-cast thin films (ca. 40 nm thick) were obtained in a Leica EM UC7 ultramicrotome [Leica EM UC7 from Leica Microsystems (Wetzlar, Germany)], and subsequently, the sections were picked up on 600-mesh copper grids. The grids were then placed in vapors of a 2% OsO_4_–water solution (Science Services, Munich, Germany)] for selective staining of the poly(butadiene) segment in order to increase the electron density through crosslinking and to improve the image contrast between PB, PS and PDMS segments.

## 3. Results and Discussion

Anionic polymerization enabled the synthesis of novel triblock terpolymers of the PS-*b*-PB_1,2_-*b*-PDMS and PB_1,2_-*b*-PS-*b*-PDMS sequences. All samples exhibited narrow dispersity indices, justifying the high standards of the living polymerization. The SEC chromatographs are presented in the Supporting Information (Figure S1a–c) and verify the absence of any by-product during the synthetic procedure of all 3 samples. For clarity reasons, the chromatographs of the PS or PB_1,2_ and intermediate diblock precursors are also given. The molecular characteristics of all blocks for the 3 triblock terpolymer samples were determined through VPO, and the results are summarized in Table 1.

Through the characteristic chemical shifts evident from the ^1^H-NMR spectra, the mass fractions of the three different segments were calculated and are also given in Table 1. The relative spectra are presented in the Supporting Information (Figure S2a–c). It is important to mention that the existence of exclusively -1,2 microstructure was verified through ^1^H-NMR, as indicated by the absence of any proton chemical shifts at 5.30 ppm. The complete absence of the two olefinic protons strongly suggests the successful synthesis of pure vinyl elastomers due to the use of 1,2-dipiperidinoethane, which is well elaborated in the literature [29]. From the molecular characterizations, it is therefore concluded that the samples showcase high molecular and compositional homogeneity, a fact that contributes to the formation of well-defined structures during morphological observations.

A DSC analysis was carried out to study the thermal behavior of the terpolymers. The thermographs are shown in the Supporting Information (Figure S3a–c). Two separate glass transition temperatures (T_g_s) of approximately −121 °C and 65 °C were recorded, corresponding exclusively to PDMS and PS blocks, respectively. The ${\bar{M}}_{n}$ values of the PB_1,2_ in almost all cases were lower than or very close (sample 1) to the entanglement molecular weight (M*_e_* ~3.8 kg/mol) [43], justifying the absence of any glass transition for the PB segments. For the semicrystalline PDMS block, additional transitions were observed at approximately −40 °C and −70 °C, indicating the melting and crystallization of the siloxane crystals, respectively, due to the increased molecular weight of the specific segment (sample 1) compared to the remaining terpolymers. The lower than expected value for the T_g_ of the PS block is extensively analyzed in the literature [41,42].

### 3.1. Structure/Properties Relationship

The as-cast OsO_4_ stained samples for approximately 30 min [44] were morphologically characterized through TEM. The unstained samples due to the similar electron densities between the PS and PB_1,2_ formed two-phase morphologies as expected [41]. Complementary experiments were conducted with SAXS to justify and verify the formation of well-developed structures. It is the very first time in the literature that the self-assembly behavior of such low molecular weight terpolymers with exclusively 100% -1,2-microstucture PB segments is studied.

The interaction parameters between the different components have been theoretically calculated at room temperature and are presented in Table 2. A thorough analysis concerning the estimation of the *χ* values based on fundamental equations from literature are provided in our previous works [30,41]. The values are critical for the interpretation of the results obtained from the morphological characterization techniques since the dissimilar driving forces promote a different self-assembly behavior for the synthesized terpolymers.

### 3.2. Sample 1 (Sample Number as Indicated in Table 1)

**Figure 1:**
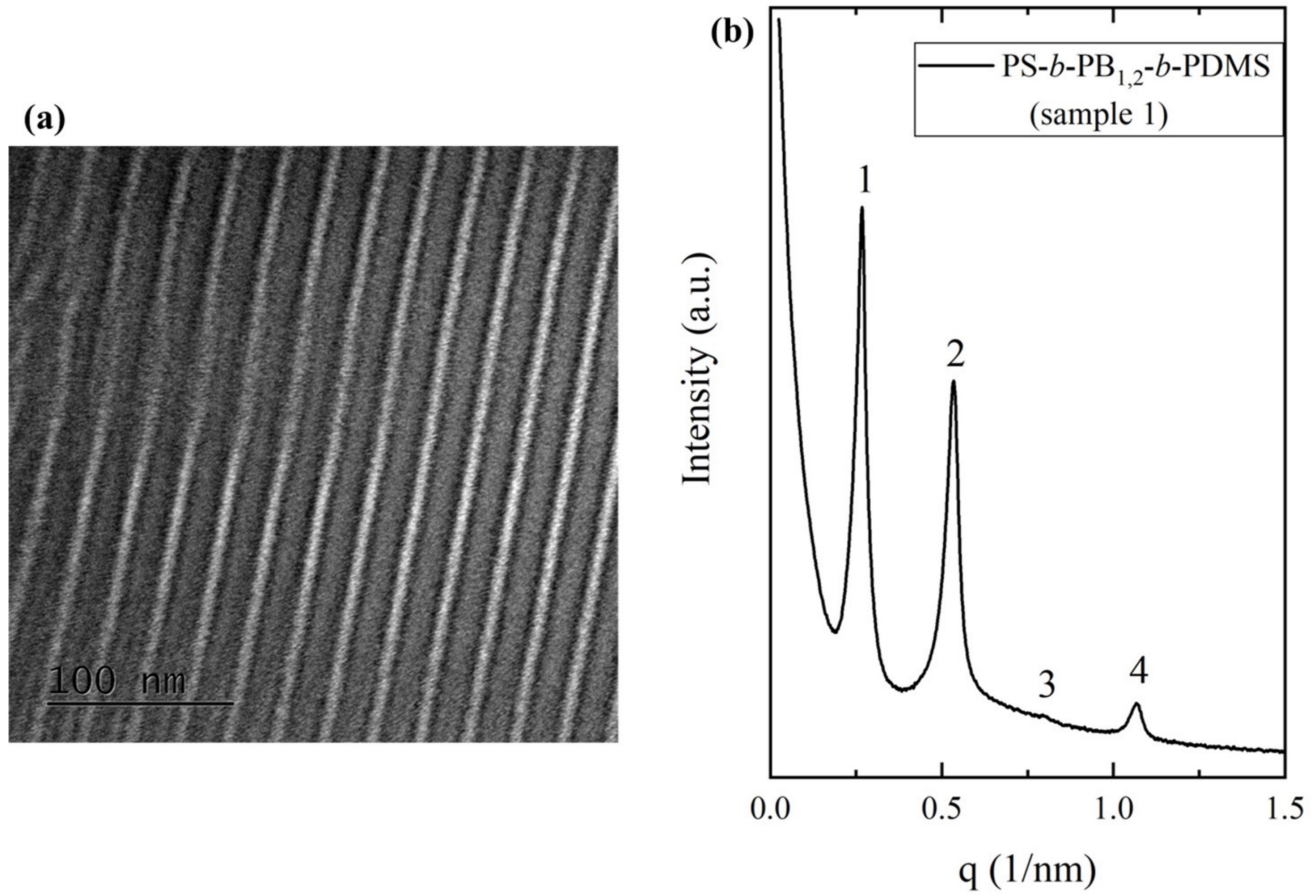
(**a**) Bright field TEM image of the PS-*b*-PB_1,2_-*b*-PDMS or sample 1, after staining with vapors of OsO_4_ for approximately 30 min. The white areas correspond to the PS, the grey to the PDMS, and the black areas to the PB_1,2_, and (**b**) the relative 1-D SAXS pattern with the characteristic reflection peaks (1:2:3:4) corresponding to lamellar morphology verifying the TEM image.

In this triblock terpolymer similar ${\bar{M}}_{n}$ values for PS as well as PB_1,2_ and increased molecular weight of the PDMS block were chosen. This strategy was followed to clarify the effect of the larger elastomeric end block on the overall morphology. The desired composition ratio for the individual segments was approximately 2/2/5, as evident in Table 1. Given that, the predicted morphology should include a PDMS matrix with the olefinic minority blocks forming a complex network. Although enthalpy-driven interactions would prevail over the entropy factors due to the major difference in molecular characteristics. The morphology obtained for this sample was three-phase four-layer lamellae, despite the non-symmetric composition ratio between the three blocks. As evident in the TEM image (Figure 1a) the white regions correspond to the PS, the black sheets to the PB_1,2_ and the gray areas to the PDMS segments. The dark color of the PB_1,2_ is attributed to the OsO_4_ staining. One could easily observe the molecular weight influence on the sheet thickness. Specifically, the dimensional approximation for the first three lamellar sheets (black/white/black) is attributed to the identical molecular weights between the two olefinic segments. The enhanced thickness of grey areas is induced by the almost doubled molecular weight of the PDMS block. The PDMS blocks due to the high incompatibility with the PS segments probably dictate the stretching of the PB_1,2_ blocks. The SAXS results further support the existence of lamellar morphology. The characteristic peak ratio of 1:2:3:4 is evident in Figure 1b, and through the first permitted reflection, the domain spacing was found to be equal to 23 nm (supporting the calculation from the TEM image of 21 nm).

### 3.3. Sample 2 (Sample Number as Indicated in Table 1)

**Figure 2:**
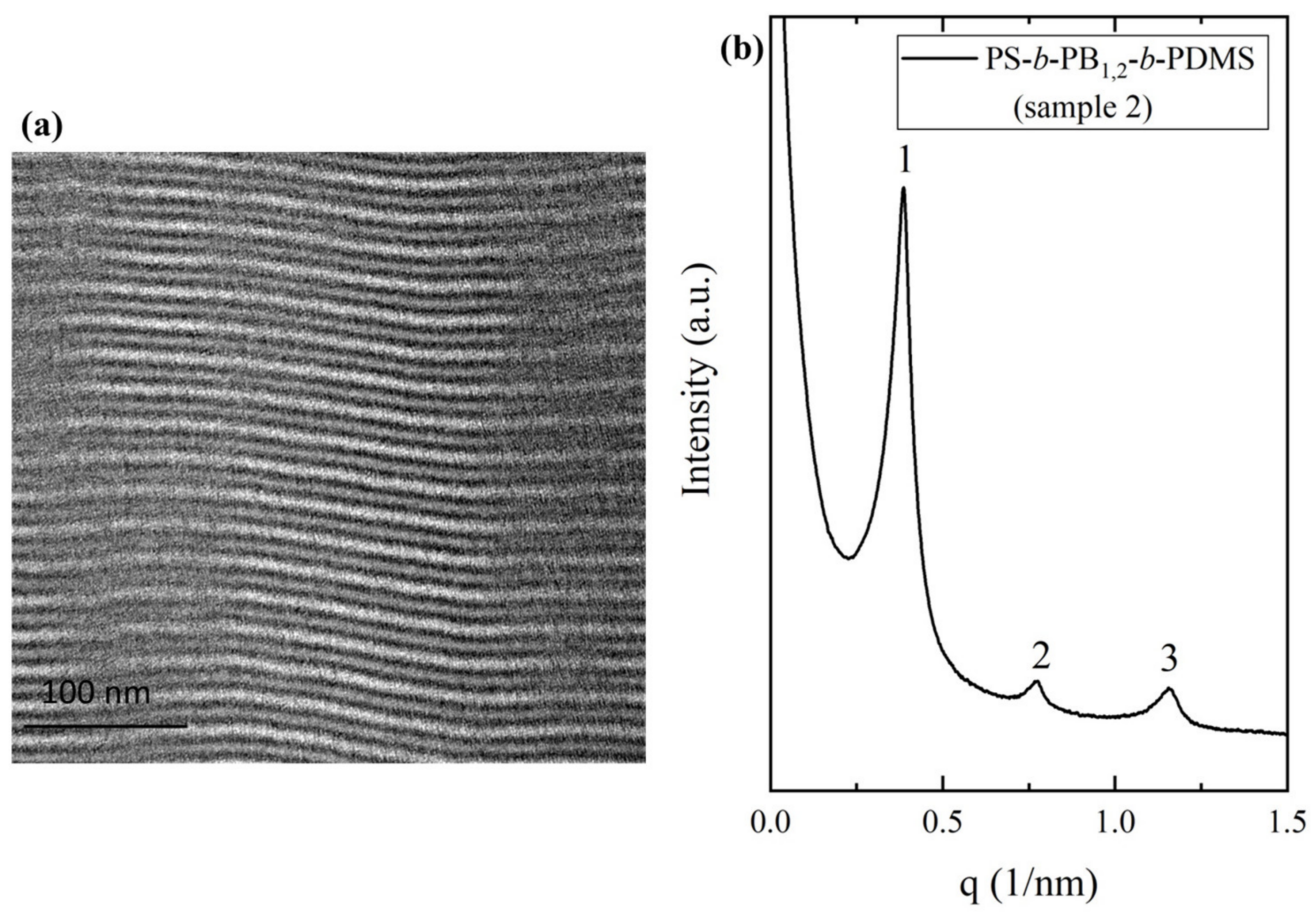
(**a**) Bright field TEM image of the PS-*b*-PB_1,2_-*b*-PDMS or sample 2, after staining with vapors of OsO_4_ for approximately 30 min. The white areas correspond to the PS, the grey to the PDMS, and the black areas to the PB_1,2_, and (**b**) the relative 1-D SAXS pattern with the characteristic reflection peaks (1:2:3) corresponding to lamellar morphology verifying the TEM image.

Taking into consideration the mass fraction ratio presented in Table 1, the specific sample shows a composition ratio equal to 4/1/5 between the three blocks. The sample adopted the three-phase four-layer lamellae morphology even though the composition ratio between the components deviates significantly from the 1/1/1 that favors the formation of lamellar sheets (as already reported for Sample 1 as well). This behavior has also been observed in our previous work [41] for the PS-*b*-PB_1,4_-*b*-PDMS sequence with approximate molecular characteristics and composition ratio. Even though the molecular weight of the blocks is quite low, especially for the intermediate elastomeric block, the system formed the structure that requires the lowest free energy. In Figure 2a, the TEM image of sample 2 is illustrated, where the black sheets correspond to the PB_1,2_ due to the enhanced electron density induced after staining, the white to the PS, and the grey to the PDMS. One would expect a different morphology to be derived from the asymmetric compositions, but the asymmetric interactions (*χ*_PS/PB_≠*χ*_PB/PDMS_≠*χ*_PS/PDMS_) have a dominant role in the formation of a less frustrated structure [45,46]. The SAXS profile corroborated the results obtained from real-space imaging (Figure 2b). Specifically, a peak ratio of 1:2:3, which is in accordance with the lamellar morphology, was observed. Through the first permitted reflection, the domain spacing was calculated to be 16 nm, which is in good proximity with the one calculated through TEM (~14 nm). The fact that the triblock terpolymer was able to self-assemble in a three-phase structure despite the low molecular characteristics of the individual blocks is of paramount importance. It is also obvious that designing high *χ* terpolymers results in different structures due to the three possible pairs of interactions.

### 3.4. Sample 3 (Sample Number as Indicated in Table 1)

**Figure 3:**
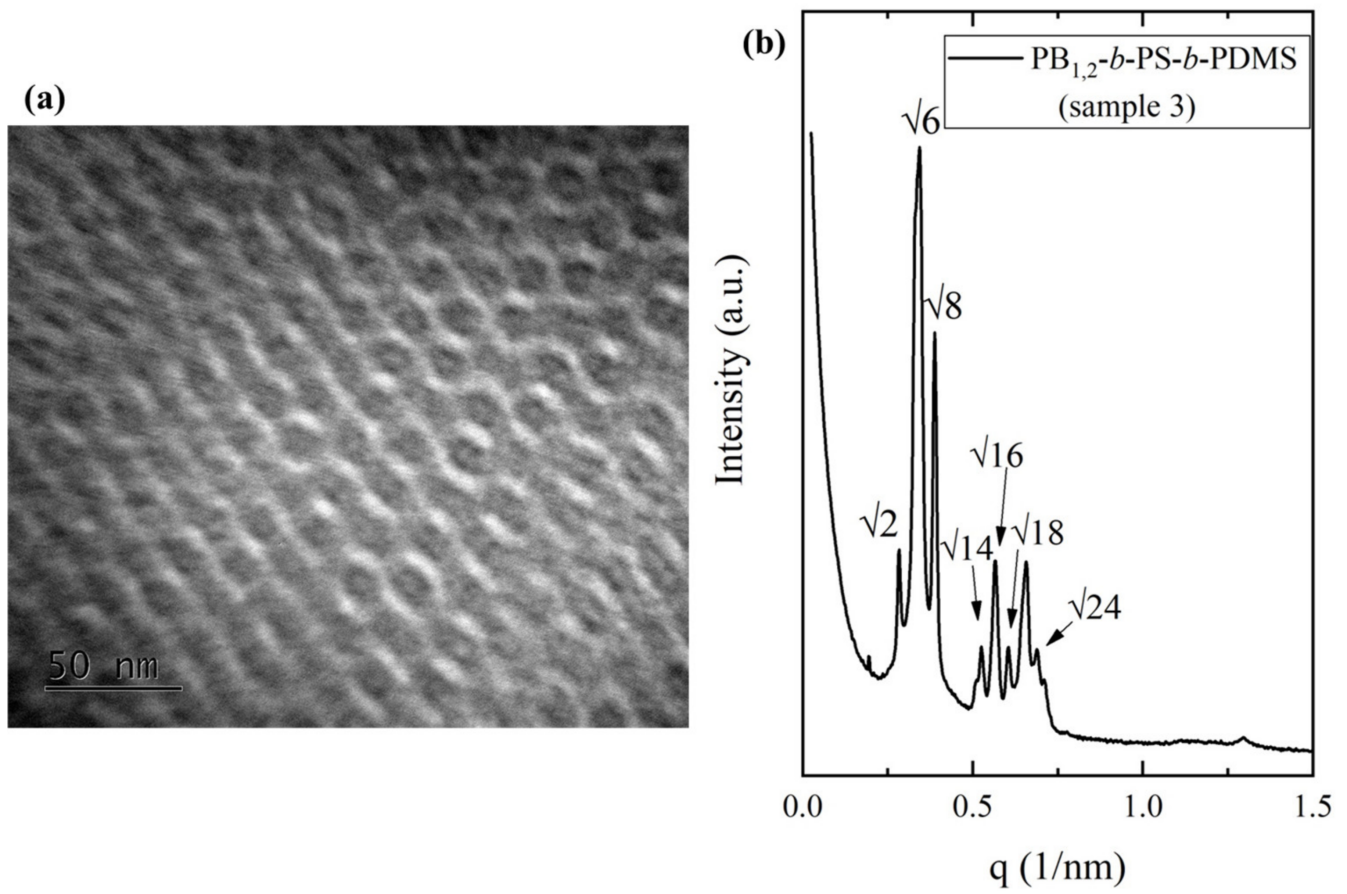
(**a**) Bright field TEM image of the PB_1,2_-*b*-PS-*b*-PDMS or sample 3, after staining with vapors of OsO_4_ for approximately 30 min. The white areas correspond to the PS, the grey to the PDMS, and the black areas to the PB_1,2_, and (**b**) the 1-D SAXS pattern with the characteristic reflections at the relative q values of √6:√8:√14:√16:√18:√24 corresponding to double gyroid morphology- verifying the TEM image.

It is well established that the ability to manipulate the block sequences in triblock terpolymers provides different nanostructures [45,46]. This extremely low molecular weight terpolymer (sample 3) showcases a composition ratio of 1/5/4, and in contrast to sample 2, the block sequence of the two segments is inversed. One might expect the formation of either three-phase, four-layer lamellae similar to the previous case or the partial mixing of PB_1,2_/PS due to the low ${\bar{M}}_{n}$ values that would eventually lead to a two-phase morphology due to the strong immiscibility between PB_1,2_/PS and PDMS. Strikingly, real-space imaging and scattering results revealed the formation of a network phase where the PS corresponds to the matrix and PB_1,2_ and PDMS constitute the two independent networks (Figure 3a). We speculate that this self-assembly behavior can be explained in terms of asymmetric interactions based on the following relationship: *χ*_PS/PDMS_ >> *χ*_PS/PB_ > *χ*_PB/PDMS_. Specifically, the most favorable interactions are between the outer segments, meaning PB_1,2_ and PDMS. The favorable enthalpic interactions in combination with the inversed sequence (compared to sample 2) induced packing frustration that allowed the formation of the cubic network phase. The results were supported by the SAXS pattern, as can be clearly identified in Figure 3b. The reflections at the relative q values of √6:√8:√14:√16:√18:√24 are in good agreement with the Ia $\bar{3}$ d space group corresponding to the double-gyroid phase. The appearance of an additional peak at the low *q* region, meaning √2, as can be observed in the relative SAXS pattern, is attributed to the distortion or deformation of the lattice [47]. The domain periodicity was calculated using the first permitted peak, which is equal to 18 nm.

### 3.5. Chemical Modification Reactions

As evident from Table 1, the total average molecular weight of PB_1,2_ was kept extremely low in all terpolymers to facilitate the physical adsorption of a very thin layer on the surface of the preferred solid substrate after the chemical modification increased its binding capacity. It would therefore be used as a polymeric brush on a corresponding surface. The chemical modification reactions were exclusively performed on the PB_1,2_ segment of each triblock terpolymer. Note that the molecular characteristics of the PS and PDMS blocks remained unchanged while the introduction of the –OH group after the modification reactions (-CH_2_-CH_2_-OH instead of -CH=CH_2_ per monomeric unit) induced an alternation on the mass fraction ratios. This alternation on the molecular characteristics is attributed to the enhanced molecular weight of the modified polybutadiene monomeric unit for the different terpolymers. The results concerning the molecular characteristics of the modified samples are summarized in Table 3.

Various techniques were employed to justify the successful chemical modification reactions, such as ^1^H-NMR (Figure S4, Supporting Information), infrared spectroscopy (Figure S5, Supporting Information), TGA (Figure S6, Supporting Information), and contact angle for modified sample 1 (sample number as indicated in Table 3). All modified samples demonstrated a similar behavior after being characterized with the different methods. The comparative results, together with the appropriate interpretation concerning modified sample 1 (sample number as indicated in Table 3) before and after the chemical modifications, are provided for better clarity.

The molecular characterization through ^1^H-NMR constitutes a reliable tool for the verification of the successful modification through the analysis of the characteristic chemical shifts. It is clear that the original chemical shifts at 5.6 ppm for the PB segment in the pristine terpolymers have been eliminated after the chemical modification, and a new characteristic shift at 2.1 ppm is evident, corresponding to the proton of the –OH group (Figure S4, Supporting Information). IR experiments further supported the ^1^H-NMR results due to the appearance of characteristic peaks at 3000–3500 cm^−1^ that are attributed to the existence of –OH groups. The IR spectra (Figure S5, Supporting Information) presented for both unmodified and modified samples are transmittance versus wave number (cm^−1^), and one can clearly observe the differentiation between the two spectra where the different vibrations are illustrated for better understanding.

DSC experiments were also carried out to study the thermal behavior of the terpolymers after the chemical modification reactions. As already discussed, in the pristine materials, in which the molecular characteristics for the PB_1,2_ were significant low (${\bar{M}}_{n}$ < 4.0 kg/mol), the glass transition temperature was absent. As a result, no alternation on the thermographs was observed, even though the physical properties of the terpolymers due to the introduction of the –OH groups are expected to differ. Additionally, TGA experiments revealed a significant weight loss at approximately 100 °C, attributed to the water molecules in the modified sample (Figure S6, Supporting Information).

**Figure 4:**
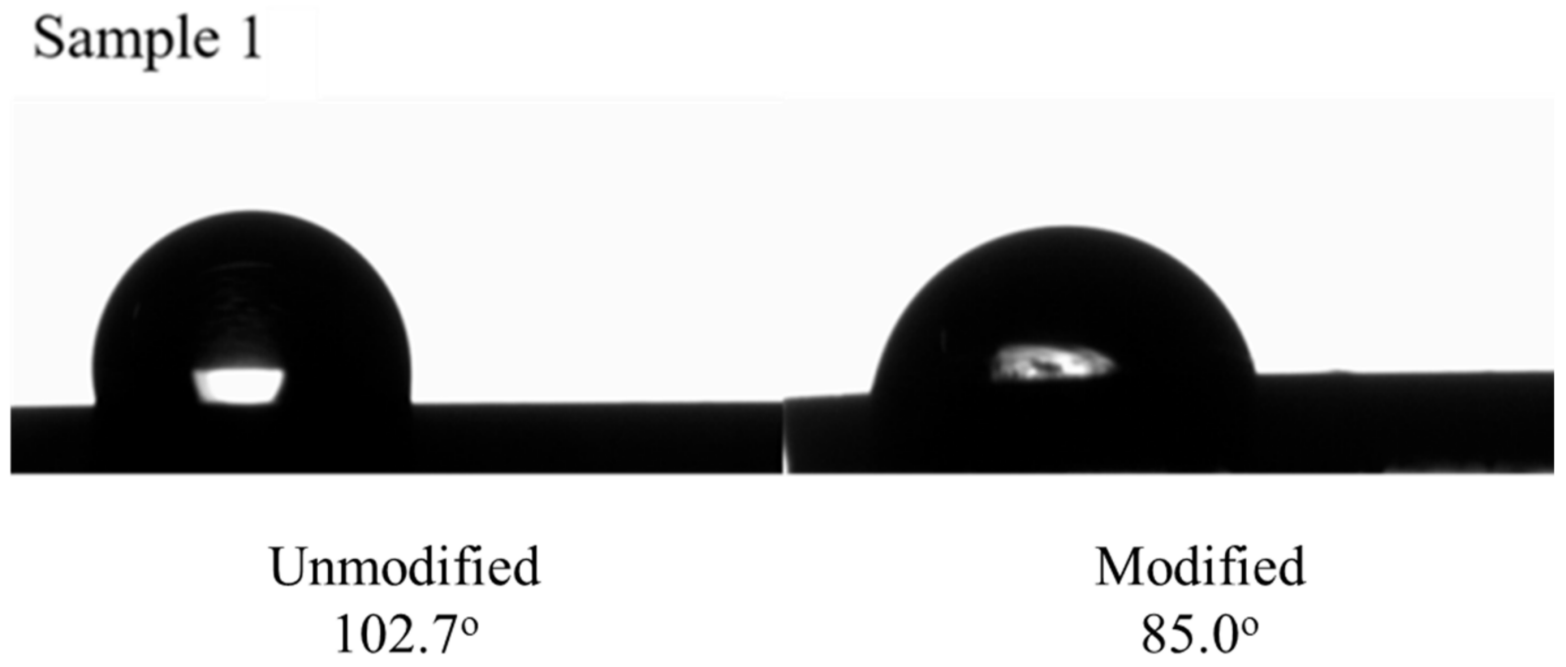
Images of water contact angles corresponding to the PS-*b*-PB_1,2_-*b*-PDMS terpolymer (sample 1). The left image corresponds to the unmodified, pristine terpolymer, and the right image to the finally modified case.

To find out whether the hydrophobicity of the modified samples is altered compared to the initial terpolymers, we have measured the surface water contact angle of the films, and the results concerning sample 1 before and after the modification are shown in Figure 4, and all data are summarized in Table 4. The results clearly indicate that the contact angle decreases (from approximately 103° to 85°) after the samples are chemically modified in all cases, a fact that is attributed to the more hydrophilic nature of the final modified terpolymer due to the incorporation of –OH groups (Figure 4).

## 4. Conclusions

Well-defined triblock terpolymers of the ABC and BAC types consisting of polystyrene (A), poly(butadiene) of exclusively (~100%) -1,2 microstructure (B) and poly(dimethylsiloxane) (C) were synthesized with anionic polymerization. The total average molecular weights were kept low to achieve as small dimensions as possible by adopting different microstructures due to the diverse composition ratios. The self-assembly behavior indicated the impact of asymmetric compositions, different interactions between the adjacent blocks, and segment sequence on the formed structures. Post-polymerization chemical modification reactions such as hydroboration and oxidation were conducted on the PB_1,2_ segments in all different terpolymers. Different molecular, thermal, and surface analysis measurements were performed to verify the successful incorporation of –OH groups in all monomeric units in the polydiene segments and therefore the preparation of polymeric brushes. The results are considered highly promising for nanotechnology applications since the necessity of homopolymer brushes during the film preparation is eliminated due to the physical absorption of the modified polydiene on the substrates.

## Figures and Tables

**Table 1:** Molecular characteristics of the synthesized linear triblock terpolymers as received from SEC, VPO, and ^1^H-NMR characterization techniques.

Sample	A-*b*-B-*b*-C	${\bar{M}}_{n}^{A}$^(a)^ (g/mol)	${\bar{M}}_{n}^{B}\;$^(a)^ (g/mol)	${\bar{M}}_{n}^{C}$^(a)^ (g/mol)	${\bar{M}}_{n}^{tot}$^(a)^ (g/mol)	Đ^SEC (b)^	*f*_A_^ (c)^	*f*_B_^ (c)^	*f*_C_^ (c)^	1,2-Microstructure ^(c)^ (%)
1	PS-*b*-PB_1,2_-*b*-PDMS	4.000	4.000	10.000	18.000	1.06	0.22	0.22	0.56	100
2	PS-*b*-PB_1,2_-*b*-PDMS	4.000	1.500	4.700	10.200	1.04	0.39	0.15	0.46	100
3	PB_1,2_-*b*-PS-*b*-PDMS	1.400	4.100	3.400	8.900	1.07	0.16	0.46	0.38	100

^a^ VPO in toluene at 45 °C. ^b^ Dispersity (Đ) calculated from SEC in ΤHF at 35 °C. ^c^ Mass fractions for the three blocks and 1,2-microstructure percentages for the PB segments calculated from ^1^H-NMR in CDCl_3_ at 25 °C.

**Table 2:** Theoretical values of Flory-Huggins interaction parameters for PS/PDMS, PS/PB_1,2_, and PB_1,2_/PDMS.

χ	PS	PB_1,2_	PDMS
PS	-	0.130	0.479
PB_1,2_	0.130	-	0.074

**Table 3:** Molecular characteristics of the modified triblock terpolymers.

Modified Sample	A-*b*-B-*b*-C	${\bar{M}}_{n}^{A}$ (g/mol)	${\bar{M}}_{n}^{B}$ (g/mol)	${\bar{M}}_{n}^{C}$ (g/mol)	${\bar{M}}_{n}^{tot}$ (g/mol)	*f*_A_	*f*_B_	*f*_C_
1	PS-*b*-PB_1,2_-*b*-PDMS (modified)	4.000	5.300	10.000	19.300	0.21	0.27	0.52
2	PS-*b*-PB_1,2_-*b*-PDMS (modified)	4.000	1.900	4.700	10.600	0.38	0.18	0.44
3	PB_1,2_-*b*-PS-*b*-PDMS (modified)	1.900	4.100	3.400	9.400	0.20	0.44	0.36

**Table 4:** Water contact angle before and after chemical modification reactions for all different terpolymers.

Sample	A-*b*-B-*b*-C	Contact Angle (Degree) Unmodified (±2°)	Contact Angle (Degree) Modified (±2°)
1	PS-*b*-PB_1,2_-*b*-PDMS	102.7	85.0
2	PS-*b*-PB_1,2_-*b*-PDMS	103.2	83.1
3	PB_1,2_-*b*-PS-*b*-PDMS	101.3	88.2

## Data Availability

The data presented in this study are available upon request from the corresponding author.

## Supplementary Materials

The following supporting information can be downloaded at: https://www.mdpi.com/article/10.3390/polym15040848/s1. Figure S1: SEC chromatographs corresponding to: (a) PS homopolymer precursor (blue), PS-*b*-PB_1,2_ intermediate diblock precursor (red) and final triblock terpolymer of the PS-*b*-PB_1,2_-*b*-PDMS type or sample 1 (black), (b) PS homopolymer precursor (blue), PS-*b*-PB_1,2_ intermediate diblock precursor (red) and final triblock terpolymer of the PS-*b*-PB_1,2_-*b*-PDMS type or sample 2 (black) and (c) (a) PB_1,2_ homopolymer precursor (blue), PB_1,2_-*b*-PS intermediate diblock precursor (red) and final triblock terpolymer of the PB_1,2_-*b*-PS-*b*-PDMS type or sample 3 (black); Figure S2: ^1^H-NMR spectra corresponding to: (a) PS-*b*-PB_1,2_-*b*-PDMS type or sample 1, (b) PS-*b*-PB_1,2_-*b*-PDMS type or sample 2 and (c) PB_1,2_-*b*-PS-*b*-PDMS type or sample 3 indicating the chemical shifts attributed to the characteristic protons of the three different monomeric units of PS, PB and PDMS respectively; Figure S3: DSC thermographs corresponding to: (a) PS-*b*-PB_1,2_-*b*-PDMS type or sample 1, (b) PS-*b*-PB_1,2_-*b*-PDMS type or sample 2, and (c) PB_1,2_-*b*-PS-*b*-PDMS type or sample 3; Figure S4: ^1^H-NMR spectra of the PS-*b*-PB_1,2_-*b*-PDMS triblock terpolymer (sample 1) before (up) and after (down) chemical modification reactions; Figure S5: FT-IR spectra of the PS-*b*-PB_1,2_-*b*-PDMS triblock terpolymer (sample 1) before (black spectrum) and after (red spectrum) chemical modification reactions; Figure S6: TGA thermograms of the PS-*b*-PB_1,2_-*b*-PDMS triblock terpolymer (sample 1) before (black spectrum) and after (red spectrum) chemical modification reactions.
