# Supplementary material for: Thermal and Bulk Properties of Triblock Terpolymers and Modified Derivatives towards Novel Polymer Brushes
Source: Polymers (Basel). 2023 Feb 8;15(4):848. doi: 10.3390/polym15040848 (PMC9965776; doi:10.3390/polym15040848)
Supplement: Supplementary file 1 [file polymers-15-00848-s001.zip › polymers-2194950-supplementary.pdf]

Thermal and Bulk Properties of Triblock Terpolymers and Modified Derivatives Towards Novel Polymer Brushes

Konstantinos Artopoiadis [1], Christina Miskaki [1], Gkreti-Maria Manesi [1], Ioannis Moutsios [1,2], Dimitrios Moschovas [1], Alexey A. Piryazev [3], Maria Karabela [1], Nikolaos E. Zafeiropoulos [1], Dimitri A. Ivanov [2,3,4] and Apostolos Avgeropoulos [1,4,*]

1 Department of Materials Science Engineering, University of Ioannina, University Campus-Dourouti, 45110 Ioannina, Greece
2 Institut de Sciences des Matériaux de Mulhouse—IS2M, CNRS UMR7361, 15 Jean Starcky, 68057 Mulhouse, France
3 Institute of Problems of Chemical Physics, Russian Academy of Sciences, Chernogolovka, 142432 Moscow, Russia
4 Faculty of Chemistry, Lomonosov Moscow State University (MSU), GSP-1, 1-3 Leninskiye Gory, 119991 Moscow, Russia
* Correspondence: aavger@uoi.gr; Tel.: +30-2651009001

a)

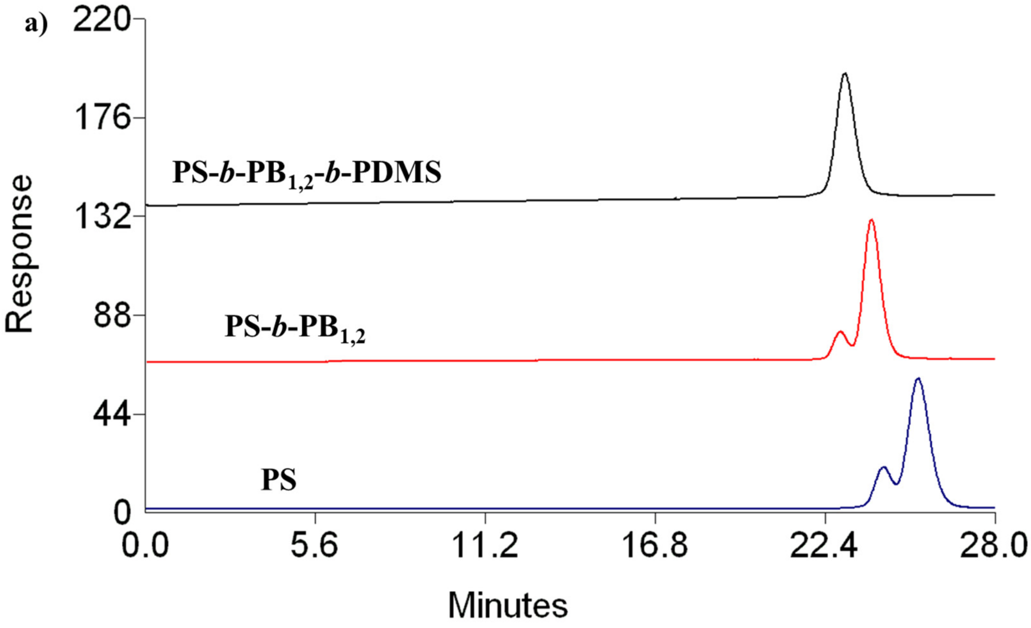

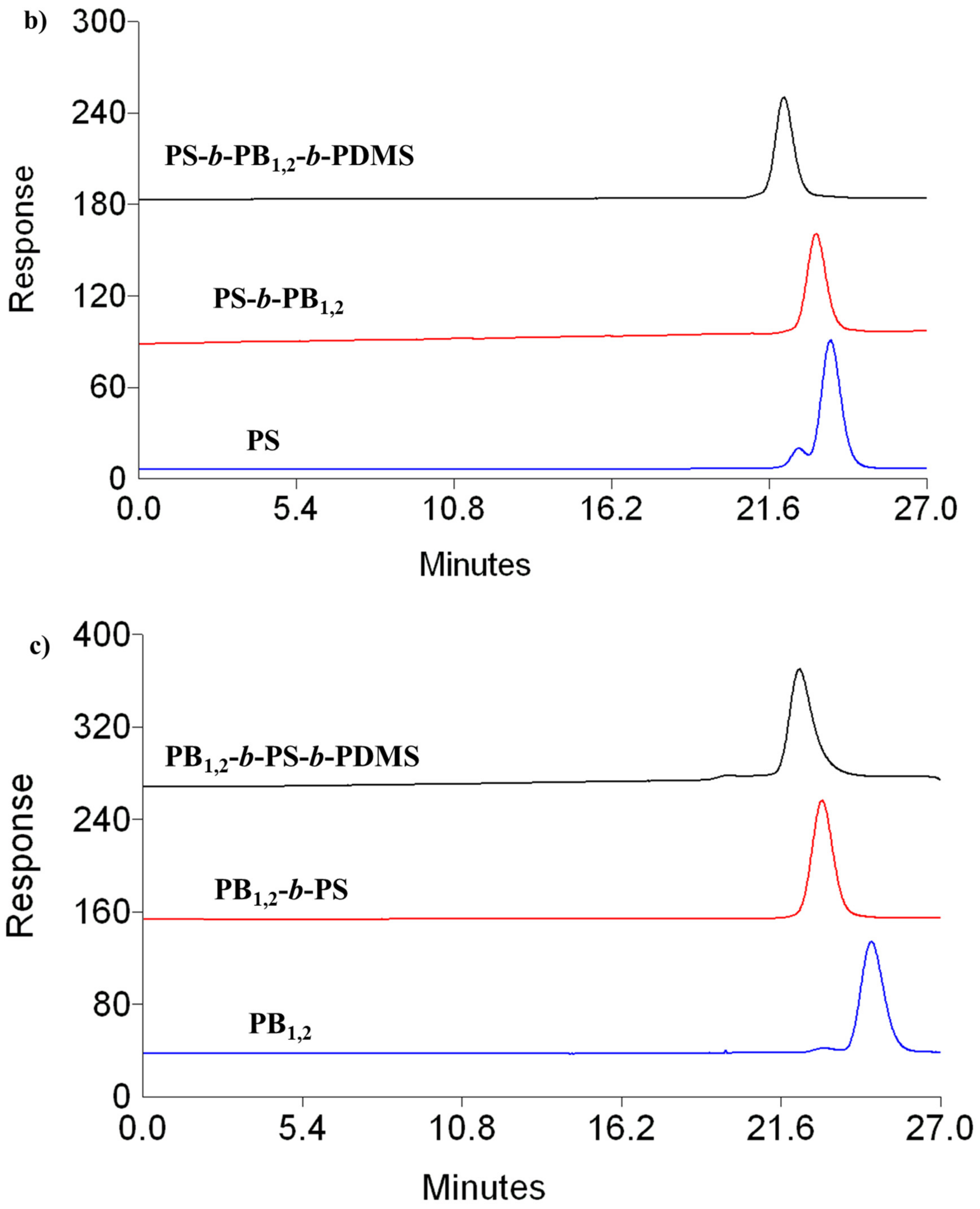

Figure S1. SEC chromatographs corresponding to: a) PS homopolymer precursor (blue), PS-*b*-$PB_{1,2}$ intermediate diblock precursor (red) and final triblock terpolymer of the PS-*b*-$PB_{1,2}$-*b*-PDMS type or sample 1 (black), b) PS homopolymer precursor (blue), PS-*b*-$PB_{1,2}$ intermediate diblock precursor (red) and final triblock terpolymer of the PS-*b*-$PB_{1,2}$-*b*-PDMS type or sample 2 (black) and c) a) $PB_{1,2}$ homopolymer precursor (blue), $PB_{1,2}$-*b*-PS intermediate diblock precursor (red) and final triblock terpolymer of the $PB_{1,2}$-*b*-PS-*b*-PDMS type or sample 3 (black).

a)

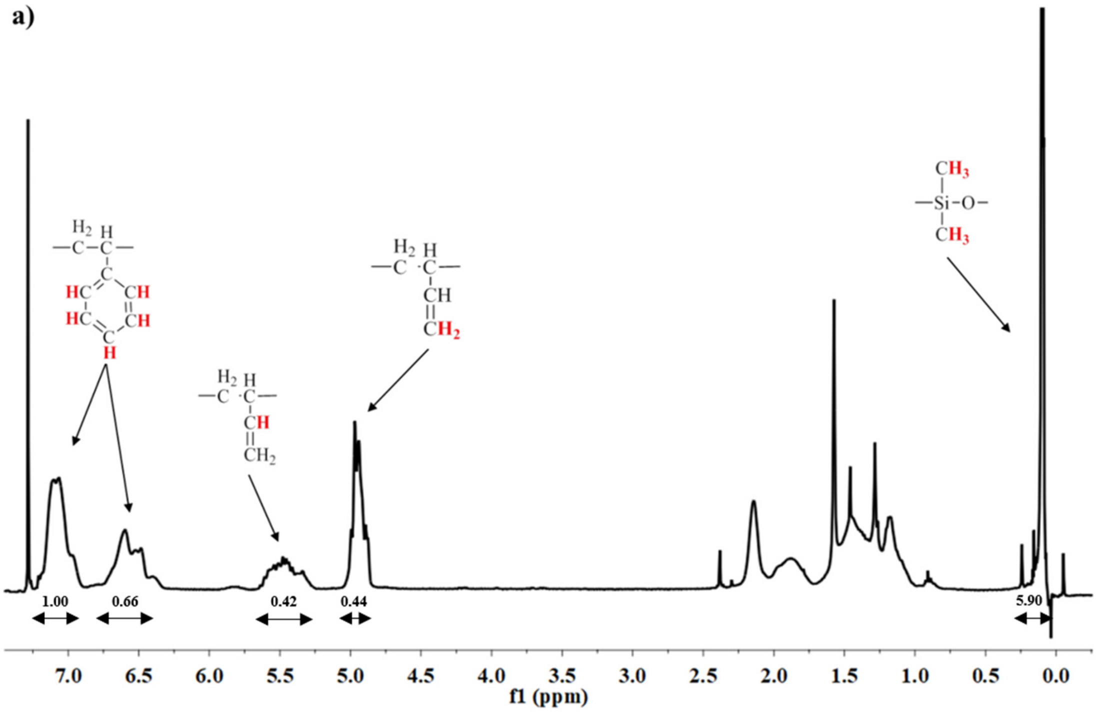

1.00
0.66
0.42
0.44
5.90
7.0 6.5 6.0 5.5 5.0 4.5 4.0 3.5 3.0 2.5 2.0 1.5 1.0 0.5 0.0
f1 (ppm)

b)

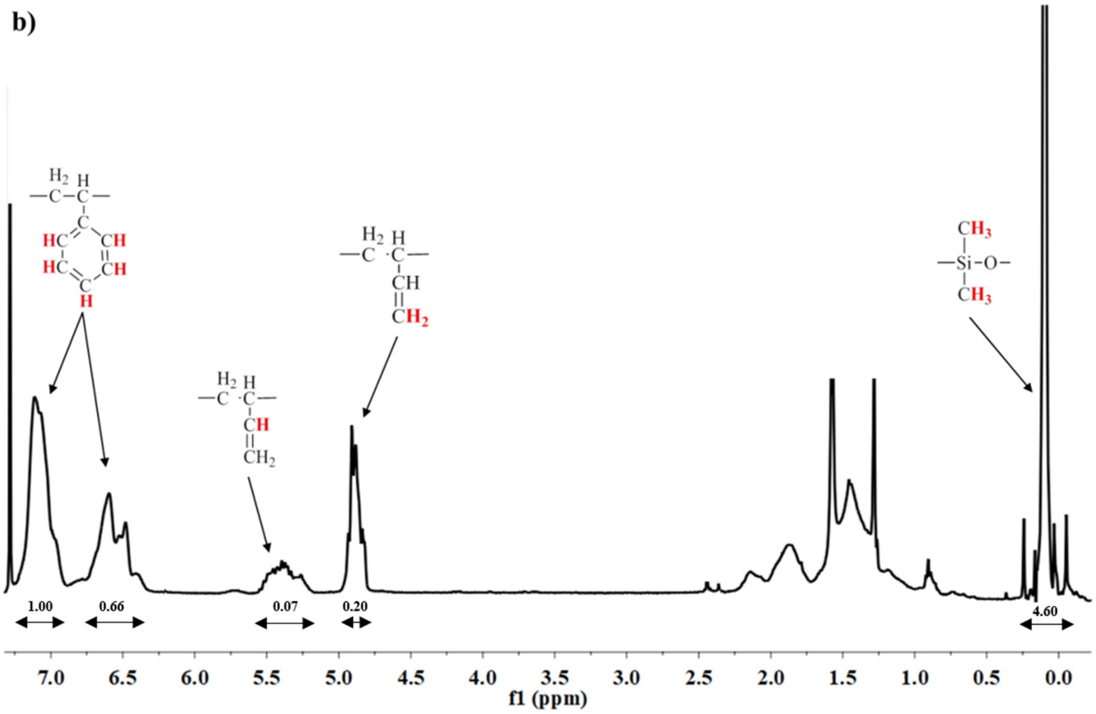

1.00
0.66
0.07
0.20
4.60
7.0 6.5 6.0 5.5 5.0 4.5 4.0 3.5 3.0 2.5 2.0 1.5 1.0 0.5 0.0
f1 (ppm)

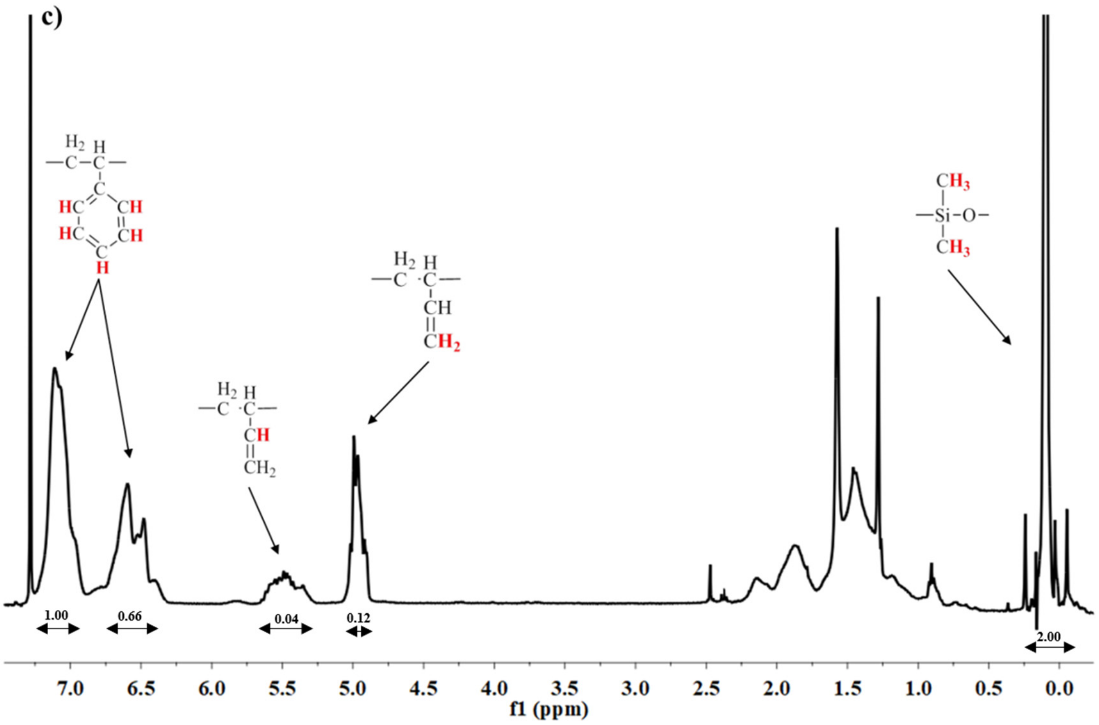

Figure S2. ^{1}H-NMR spectra corresponding to: (a) PS-*b*-$PB_{1,2}$-*b*-PDMS type or sample 1, (b) PS-*b*-$PB_{1,2}$-*b*-PDMS type or sample 2 and (c) $PB_{1,2}$-*b*-PS-*b*-PDMS type or sample 3, indicating the chemical shifts attributed to the characteristic protons of the three different monomeric units of PS, PB and PDMS respectively.

The chemical shifts corresponding to specific protons in the monomeric units of PS, $PB_{1,2}$ and PDMS segments observed in the ^{1}H-NMR spectra further verified the successful synthesis. The chemical shifts in the region 0-0.2 ppm are assigned to the six (6) protons of the two methyl groups in the monomeric units of PDMS. The chemical shifts in 5.0 ppm (2 protons) and 5.6 ppm (1 proton) are attributed to the three (3) protons of the methyl groups in the monomeric units of $PB_{1,2}$ microstructure and in the region 6.7-7.7 ppm the chemical shifts to the five (5) protons of the aromatic ring in the monomeric units of PS. The shifts are in good accordance with the ones reported in the literature.

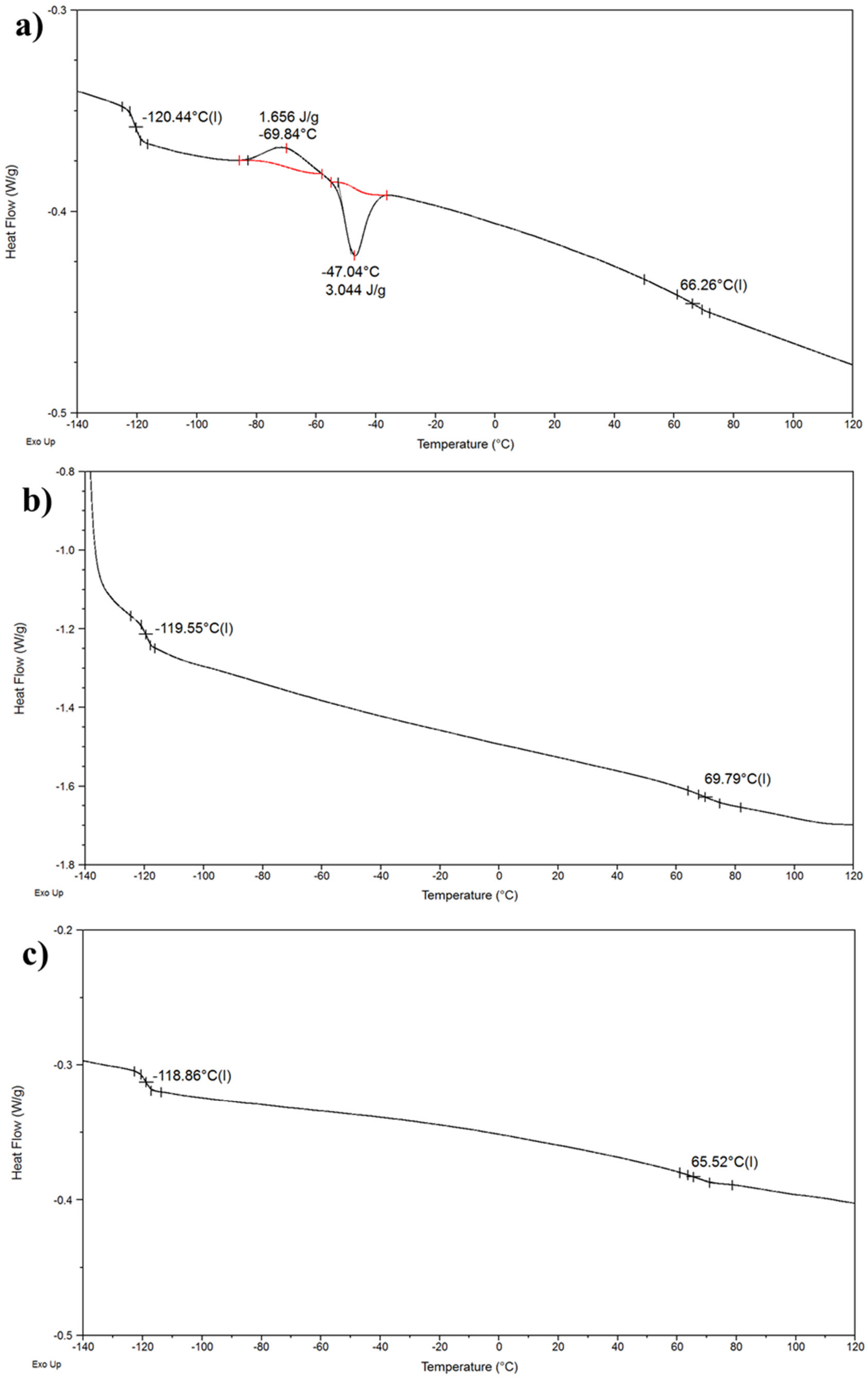

Figure S3. DSC thermographs corresponding to: (a) PS-*b*-PB$_{1,2}$-*b*-PDMS type or sample 1, (b) PS-*b*-PB$_{1,2}$-*b*-PDMS type or sample 2 and (c) PB$_{1,2}$-*b*-PS-*b*-PDMS type or sample 3.

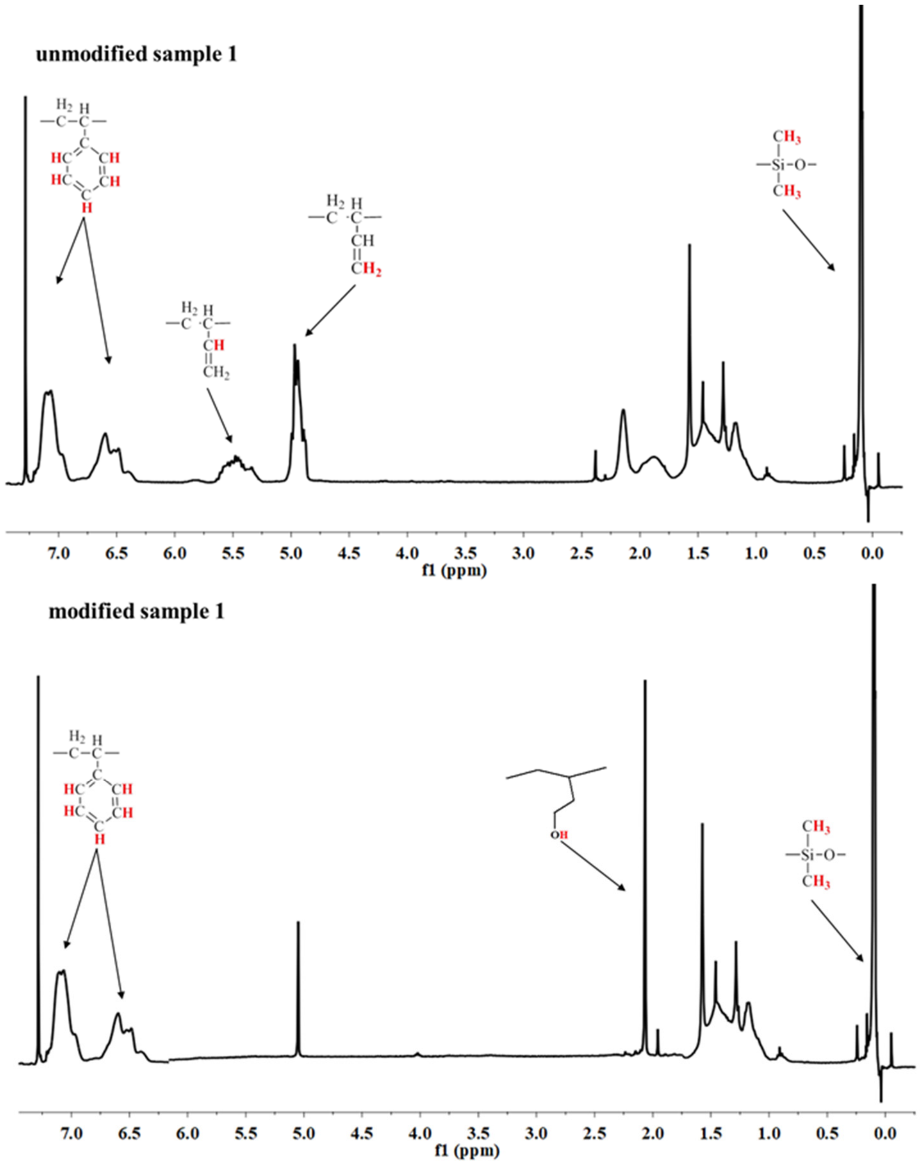

Figure S4. ¹H-NMR spectra of the PS-*b*-$PB_{1,2}$-*b*-PDMS triblock terpolymer (sample 1) before (up) and after (down) chemical modification reactions.

As already described the chemical shifts in 5.0 ppm (2 protons) and 5.6 ppm (1 proton) are attributed to the three (3) protons of the methyl groups in the monomeric units of $PB_{1,2}$ microstructure. After performing the chemical modification reactions the chemical shifts disappeared due to the introduction of –OH side groups. The proton chemical shift located at 2.1 ppm indicates the successful modification reaction.

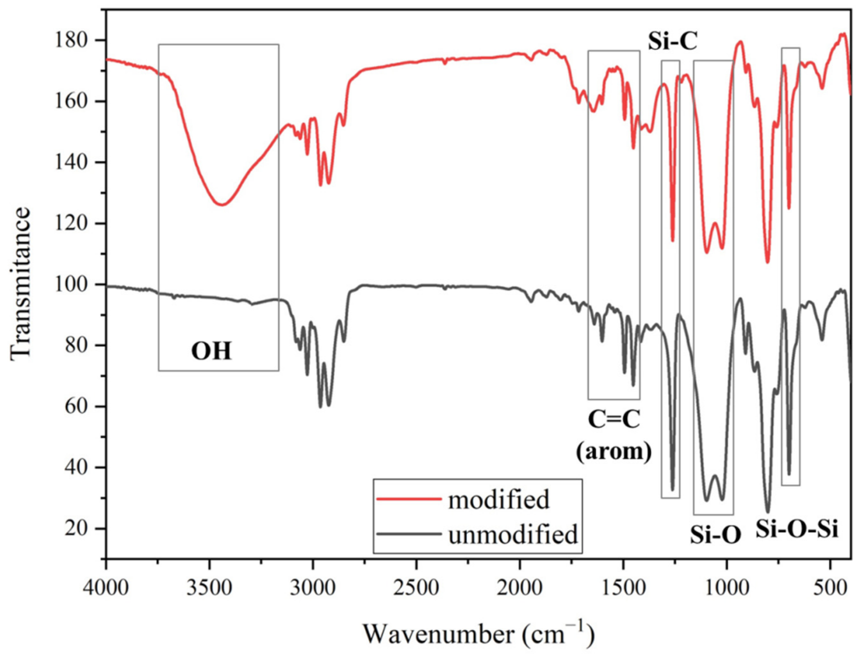

Figure S5. FT-IR spectra of the PS-*b*-$PB_{1,2}$-*b*-PDMS triblock terpolymer (sample 1) before (black spectrum) and after (red spectrum) chemical modification reactions.

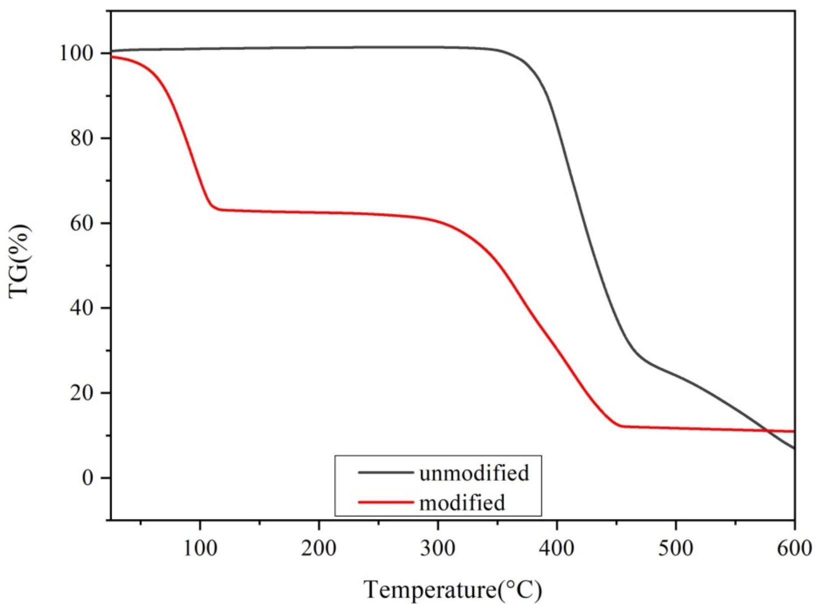

Figure S6. TGA thermograms of the PS-*b*-$PB_{1,2}$-*b*-PDMS triblock terpolymer (sample 1) before (black spectrum) and after (red spectrum) chemical modification reactions.
